# Lymph node evaluation for endometrial hyperplasia: a nationwide analysis of minimally invasive hysterectomy in the ambulatory setting

**DOI:** 10.1007/s00464-023-10081-2

**Published:** 2023-05-08

**Authors:** Koji Matsuo, Katharine M. Ciesielski, Rachel S. Mandelbaum, Matthew W. Lee, Neda D. Jooya, Lynda D. Roman, Jason D. Wright

**Affiliations:** 1 Division of Gynecologic Oncology, Department of Obstetrics and Gynecology, University of Southern California, 2020 Zonal Avenue, IRD 520, Los Angeles, CA 90033 USA; 2 Norris Comprehensive Cancer Center, University of Southern California, Los Angeles, CA USA; 3 Division of Reproductive Endocrinology & Infertility, Department of Obstetrics and Gynecology, University of Southern California, Los Angeles, CA USA; 4 Division of Gynecologic Oncology, Department of Obstetrics and Gynecology, Columbia University College of Physicians and Surgeons, New York, NY USA

**Keywords:** Endometrial hyperplasia, Hysterectomy, Minimally invasive, Ambulatory, Same day surgery, Lymph node evaluation

## Abstract

**Background:**

Given the possibility of occult endometrial cancer where nodal status confers important prognostic and therapeutic data, role of lymph node evaluation at hysterectomy for endometrial hyperplasia is currently under active investigation. The objective of the current study was to examine the characteristics related to lymph node evaluation at the time of minimally invasive hysterectomy when performed for endometrial hyperplasia in an ambulatory surgery setting.

**Methods:**

The Healthcare Cost and Utilization Project's Nationwide Ambulatory Surgery Sample was retrospectively queried to examine 49,698 patients with endometrial hyperplasia who underwent minimally invasive hysterectomy from 1/2016 to 12/2019. A multivariable binary logistic regression model was fitted to assess the characteristics related to lymph node evaluation at hysterectomy and a classification tree model with recursive partitioning analysis was constructed to examine the utilization pattern of lymph node evaluation.

**Results:**

Lymph node evaluation was performed in 2847 (5.7%) patients. In a multivariable analysis, (i) patient factors with older age, obesity, high census-level household income, and large fringe metropolitan, (ii) surgical factors with total laparoscopic hysterectomy and recent year surgery, (iii) hospital parameters with large bed capacity, urban setting, and Western U.S. region, and (iv) histology factor with presence of atypia were independently associated with increased utilization of lymph node evaluation at hysterectomy (all, *P* < 0.05). Among those independent factors, presence of atypia exhibited the largest association for lymph node evaluation (adjusted odds ratio 3.75, 95% confidence interval 3.39–4.16). There were 20 unique patterns of lymph node evaluation based on histology, hysterectomy type, patient age, year of surgery, and hospital bed capacity, ranging from 0 to 20.3% (absolute rate difference, 20.3%).

**Conclusion:**

Lymph node evaluation at the time of minimally invasive hysterectomy for endometrial hyperplasia in the ambulatory surgery setting appears to be evolving with large variability based on histology type, hysterectomy modality, patient factors, and hospital parameters, warranting a consideration of developing clinical practice guidelines.

**Supplementary Information:**

The online version contains supplementary material available at 10.1007/s00464-023-10081-2.

Endometrial hyperplasia is a premalignant precursor of endometrial cancer and is characterized by the presence of disorganized proliferative endometrial glands [1, 2]. Endometrial hyperplasia is associated with unopposed effect of estrogen exposure, and the incidence is estimated to be several fold higher than that of endometrial cancer [3].

Given the risk of progression to endometrial cancer (1–29%), hysterectomy is the treatment of choice for patients with endometrial hyperplasia who have completed childbearing [1, 2, 4]. Occult endometrial cancer can be identified in approximately 40% of hysterectomy specimens in the setting of preoperative endometrial hyperplasia with atypia (42.6%) [5]. In cases in which occult endometrial cancer is identified, regional lymph node metastasis is estimated to occur in 1.6–2.1% of patients with a preoperative diagnosis of endometrial hyperplasia with atypia [6]. In this population the status of the regional lymph nodes confers important prognostic and therapeutic data [7]. Importantly, the reproducibility of endometrial hyperplasia with atypia across pathologists is poor (38%) and adenocarcinoma is often underestimated [8]. Approximately, 30% of occult endometrial cancers meet the criteria for lymph node evaluation (28%) [9].

The risk of occult endometrial cancer in patients with endometrial hyperplasia has sparked interest in performance of lymph node evaluation at the time of hysterectomy for endometrial hyperplasia. A recent analysis of the Premier Perspective Healthcare Database found that utilization of lymph node evaluation at hysterectomy for endometrial hyperplasia with atypia increased from 0.8 in 2012 to 14.0% in 2018 [10]. Another recent national-level analysis of inpatient hysterectomy for endometrial hyperplasia using the National Inpatient Sample showed increasing utilization of lymph node evaluation for endometrial hyperplasia with atypia (6.3% to 13.4%) and without atypia (1.2% to 4.4%) from 2016 to 2019 [11].

Given that the modality of hysterectomy is shifting from abdominal to minimally invasive and from the inpatient to ambulatory setting in the USA [12, 13], examination of the rate of lymph node evaluation during minimally invasive hysterectomy in the ambulatory setting for endometrial hyperplasia is of interest. The objective of this study was to examine the characteristics related to lymph node evaluation at the time of minimally invasive hysterectomy when performed for endometrial hyperplasia in an ambulatory surgical setting.

## Materials and methods

### Data

This is a retrospective cohort study querying the Healthcare Cost and Utilization Project’s Nationwide Ambulatory Surgery Sample (NASS) [14]. NASS is the largest all-payer database for ambulatory surgery in the USA. The program collects information for ambulatory surgery performed in hospital-owned facilities. In 2019, nearly 9 million encounters, estimating 11.8 million encounters nationally, were collected across 2958 centers. The data capture schema of NASS represents approximately 68% of ambulatory surgeries in U.S. hospital-owned facilities. The University of Southern California Institutional Review Board deemed this study exempt due to the use of publicly available, deidentified data.

### Eligibility

The study population was patients with a diagnosis of endometrial hyperplasia who underwent minimally invasive hysterectomy between January 2016 and December 2019. Endometrial hyperplasia was based on an International Classification of Disease 10th revision (ICD-10) code of N85.0 per a prior analysis (Supplementary Table S1) [15]. In this study, minimally invasive hysterectomy was defined as total laparoscopic hysterectomy (TLH), laparoscopic-assisted vaginal hysterectomy (LAVH), and total vaginal hysterectomy (TVH). The American Medical Association's Current Procedural Terminology (CPT) codes for these hysterectomies followed a prior study (Supplementary Table S1) [13]. These CPT codes were not distinguishable for robotic-assisted surgery.

Exclusion criteria included other hysterectomy types (supracervical hysterectomy and abdominal hysterectomy) and the presence of gynecologic malignancy (uterine cancer, cervical cancer, and ovarian cancer). Supracervical hysterectomy was excluded as this is not a standard surgical procedure for endometrial hyperplasia. Abdominal hysterectomy was excluded as this approach is generally performed in inpatient settings and was rarely performed in this cohort (< 1%). The exclusion of invasive malignancy was to ensure that lymph node evaluation at hysterectomy was most likely performed for endometrial hyperplasia.

### Exposure assignment

The study population was grouped based on the performance of lymph node evaluation at the time of hysterectomy. CPT and Clinical Classification Software (CCS) codes were used to identify patients who had lymph node evaluation (Supplementary Table S1). The CPT codes chosen were based on prior studies [16, 17]. CCS code was provided per the Healthcare Cost and Utilization Project [18]. Patients who had any one of these codes were assigned as lymph node evaluation and those who did not have any of these codes were assigned as no lymph node evaluation in this study.

### Outcome measures

We analyzed the patterns of utilization of lymph node evaluation at hysterectomy for endometrial hyperplasia as well as clinical and hospital characteristics associated with nodal evaluation.

### Study covariates

Among the eligible cases, patient demographics, hospital parameters, surgical information, and histologic data were abstracted from the database.

Patient demographics included age at surgery (< 40, 40–59, and ≥ 60 years) grouped per a prior study [19], year of encounter (2016, 2017, 2018, and 2019), primary expected payer (Medicare, Medicaid, private including HMO, self-pay, no charge, and other), median household income for patient’s ZIP code (every quartile), patient location (large central metropolitan, large fringe metropolitan, medium metropolitan, small metropolitan, micropolitan, and not metropolitan or micropolitan counties), obesity (yes or no), and Charlson comorbidity index (0, 1, 2, and ≥ 3) calculated as previously described [15, 20].

Hospital parameters included hospital bed capacity (small, mid, and large), hospital location and teaching setting (rural, urban non-teaching, and urban teaching), and hospital region (Northeast, Midwest, South, and West). Surgical information included mode of hysterectomy (TLH, LAVH, and TVH). Histology data included endometrial hyperplasia with atypia and endometrial hyperplasia without atypia.

### Statistical analysis

The first step of analysis was to identify the independent characteristics related to lymph node evaluation at the time of hysterectomy for endometrial hyperplasia. A multivariable binary logistic regression model was fitted for analysis, and all the measured covariates were entered in the final modeling. The effect size for lymph node evaluation was expressed with adjusted odds ratio (aOR) with a corresponding 95% confidence interval (CI).

The second step of analysis was to evaluate the utilization pattern of lymph node evaluation at the time of hysterectomy for endometrial hyperplasia. A classification tree was constructed with recursive partitioning analysis fitting the chi-square automatic interaction detector method with stopping rule of 3 maximum layers. In each identified pattern, the utilization rate of lymph node evaluation was calculated.

Sensitivity analyses included evaluation of the study cohort stratified by histologic type (endometrial hyperplasia with or without atypia). The weighted values for national estimates provided by the NASS program were utilized for statistical analysis. Statistical interpretation was based on a two-tailed hypothesis and a *P* < 0.05 was considered statistically significant. Cases with missing information were grouped as one category in each variable. IBM SPSS Statistics (version 28.0, Armonk, NY, USA) was used for all analysis. The STROBE reporting guidelines were followed to summarize the performance of the cohort study [21].

## Results

A total of 49,698 patients who underwent minimally invasive hysterectomy in the ambulatory setting from 2016 to 2019 were identified. The cohort-level characteristics are shown in Table 1. The median age was 53 years (interquartile range 46–62). Patients were most commonly privately insured (67.8%) and underwent surgery at large (58.7%) and urban teaching (69.9%) centers. The majority had TLH (*n* = 36,511, 73.5%), followed by LAVH (*n* = 9192, 18.5%) and TVH (*n* = 3995, 8.0%).

**Table 1:** Patient demographics

Characteristic	No. (%)*	LN^†^	*P* value
All	49,698 (100)	5.7	
Age (years)			< 0.001
< 40	5421 (10.9)	2.5	
40–59	28,930 (58.2)	5.1	
≥ 60	15,344 (30.9)	8.0	
Unknown	**	0	
Year			< 0.001
2016	12,506 (25.2)	3.6	
2017	12,712 (25.6)	4.7	
2018	11,742 (23.6)	4.5	
2019	12,739 (25.6)	10.0	
Primary expected payer			< 0.001
Medicare	9597 (19.3)	7.6	
Medicaid	4259 (8.6)	4.0	
Private including HMO	33,678 (67.8)	5.4	
Self-pay	936 (1.9)	6.5	
No charge	73 (0.1)	**	
Other	1089 (2.2)	5.3	
Unknown	64 (0.1)	**	
Household income			< 0.001
QT1 (lowest)	10,822 (21.8)	4.7	
QT2	13,312 (26.8)	5.3	
QT3	13,254 (26.7)	5.7	
QT4 (highest)	11,702 (23.5)	7.2	
Unknown	610 (1.2)	6.9	
Patient location			< 0.001
Large central metropolitan	12,419 (25.0)	6.2	
Large fringe metropolitan	12,440 (25.0)	6.7	
Medium metropolitan	10,984 (22.1)	5.7	
Small metropolitan	4989 (10.0)	5.0	
Micropolitan	5247 (10.6)	4.0	
Not metropolitan or micropolitan	3581 (7.2)	4.4	
Unknown	39 (0.1)	**	
Obesity			< 0.001
No	33,874 (68.2)	5.0	
Yes	15,824 (31.8)	7.3	
Charlson comorbidity index			< 0.001
0	34,258 (68.9)	5.3	
1	10,310 (20.7)	6.4	
2	3491 (7.0)	7.0	
≥ 3	1638 (3.3)	8.2	
Hospital bed capacity			< 0.001
Small	3983 (8.0)	2.1	
Mid	16,529 (33.3)	4.2	
Large	29,187 (58.7)	7.1	
Hospital location/teaching			< 0.001
Rural	4479 (9.0)	1.2	
Urban non-teaching	10,499 (21.1)	4.1	
Urban teaching	34,720 (69.9)	6.8	
Hospital region			< 0.001
Northeast	8122 (16.3)	5.3	
Midwest	13,503 (27.2)	5.3	
South	20,240 (40.7)	5.4	
West	7832 (15.8)	7.8	
Hysterectomy modality			< 0.001
TLH	36,511 (73.5)	7.2	
LAVH	9192 (18.5)	2.3	
TVH	3995 (8.0)	0	
Histology type			< 0.001
Non-atypia	19,552 (39.3)	2.5	
Atypia	19,654 (39.5)	11.0	
NOS	10,492 (21.1)	1.9	

Total number may not be 49,698 due to the weighted model

*LN* lymph node evaluation, *QT* quartile, *TLH* total laparoscopic hysterectomy, *LAVH* laparoscopy-assisted vaginal hysterectomy, *TVH* total vaginal hysterectomy, *NOS* not otherwise specified

*Percentage per column

**Small number suppressed per HCUP guidelines (1–10)

^†^LN rate (%) per row

**Fig. 1:**
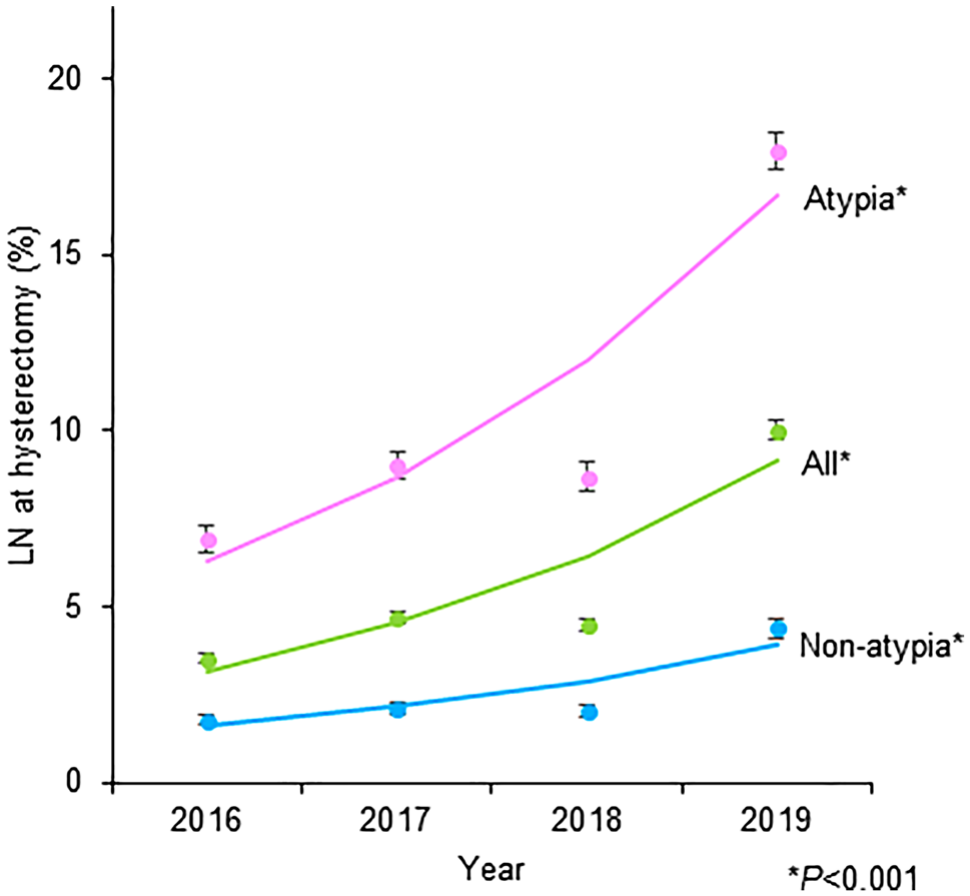
Trends of lymph nodal evaluation at hysterectomy. The utilization rates of lymph node evaluation at the time of hysterectomy for endometrial hyperplasia is shown per calendar year. *Cochran–Armitage trend test for *P* value. Dots represent observed values and bars represent standard error. Bold lines represent the modeled estimates. The *Y*-axis is truncated to 0–20% for visibility

During the study period, 2,847 (5.7%) patients had lymph node evaluation at the time of hysterectomy. The utilization of lymph node evaluation increased from 3.6% in 2016 to 10.0% in 2019 (*P* *trend* < 0.001; Fig. 1).

In a univariable analysis (Table 1), all the measured covariates were statistically associated with lymph node evaluation (all, *P* < 0.001). In a multivariable analysis (Table 2), (i) patient factors including older age, obesity, higher census-level household income, and large fringe metropolitan location, (ii) surgical factors with TLH and more recent year of surgery, (iii) hospital parameters including large bed capacity, urban setting, and Western U.S. region, and (iv) histologic factors including the presence of atypia were independently associated with increased utilization of lymph node evaluation at hysterectomy (all, *P* < 0.05).

**Table 2:** Multivariable analysis for lymph node evaluation

Characteristic	aOR (95% CI)	*P* value
Age (years)		
< 40	1 (reference)	
40–59	1.87 (1.55–2.25)	< 0.001
≥ 60	2.51 (2.07–3.06)	< 0.001
Unknown	n/a	0.999
Year		
2016	1 (reference)	
2017	1.28 (1.13–1.46)	< 0.001
2018	1.17 (1.03–1.34)	0.018
2019	2.65 (2.36–2.97)	< 0.001
Primary expected payer		
Medicare	1 (reference)	
Medicaid	0.96 (0.79–1.16)	0.681
Private including HMO	0.96 (0.86–1.07)	0.456
Self-pay	1.13 (0.85–1.51)	0.401
No charge	1.35 (0.48–3.77)	0.573
Other	1.04 (0.78–1.40)	0.771
Unknown	0.33 (0.06–1.81)	0.204
Household income		
QT1 (lowest)	1 (reference)	
QT2	1.13 (1.00–1.27)	0.061
QT3	1.06 (0.93–1.20)	0.406
QT4 (highest)	1.22 (1.07–1.39)	0.004
Unknown	1.40 (0.99–1.99)	0.058
Patient location		
Large central metropolitan	0.76 (0.69–0.85)	< 0.001
Large fringe metropolitan	1 (reference)	
Medium metropolitan	0.90 (0.80–1.01)	0.086
Small metropolitan	0.97 (0.83–1.14)	0.729
Micropolitan	1.00 (0.84–1.20)	0.998
Not metropolitan or micropolitan	1.07 (0.88–1.30)	0.523
Unknown	0.38 (0.07–2.05)	0.263
Obesity		
No	1 (reference)	
Yes	1.15 (1.06–1.25)	0.001
Charlson comorbidity index		
0	1 (reference)	
1	1.00 (0.91–1.11)	0.932
2	1.07 (0.93–1.24)	0.340
≥ 3	1.01 (0.83–1.23)	0.895
Hospital bed capacity		
Small	1 (reference)	
Mid	1.08 (0.84–1.37)	0.554
Large	1.55 (1.22–1.99)	< 0.001
Hospital location/teaching		
Rural	1 (reference)	
Urban non-teaching	2.79 (2.04–3.83)	< 0.001
Urban teaching	2.93 (2.14–4.02)	< 0.001
Hospital region		
Northeast	0.72 (0.64–0.81)	< 0.001
Midwest	0.95 (0.86–1.05)	0.312
South	1 (reference)	
West	1.36 (1.21–1.53)	< 0.001
Hysterectomy modality		
TLH	2.54 (2.20–2.94)	< 0.001
LAVH	1 (reference)	
TVH	n/a	0.993
Histology type		
Non-atypia	1 (reference)	
Atypia	3.75 (3.39–4.16)	< 0.001
NOS	0.74 (0.63–0.88)	0.001

A binary logistic regression model for multivariable analysis. All the listed covariates were entered in the modeling

*aOR* adjusted odds ratio, *CI* confidence interval, *QT* quartile, *TLH* total laparoscopic hysterectomy, *LAVH* laparoscopy-assisted vaginal hysterectomy, *TVH* total vaginal hysterectomy, *NOS* not otherwise specified

Among those independent factors, the presence of atypia exhibited the strongest association for lymph node evaluation (aOR compared to non-atypia 3.75). This was followed by surgery at urban teaching hospitals (aOR compared to rural 2.93) and urban non-teaching hospitals (aOR compared to rural 2.79), surgery in 2019 (aOR compared to 2016 2.65), age ≥ 60 years (aOR compared to < 40 years 2.51), and TLH (aOR compared to LAVH 2.54).

Utilization patterns of lymph node evaluation were then examined (Table 3 and Supplementary Fig. S1). The analysis identified 20 unique patterns of lymph node evaluation based on histology, hysterectomy type, patient age, year of surgery, and hospital bed capacity. In this 3-layer classification tree, histologic type was the cohort allocator of the first layer, followed by hysterectomy type, year of surgery, and age in the second layer (Supplementary Fig. S1).

**Table 3:** Classification tree model for nodal evaluation (all cases)

Pattern^‡^	Atypia	Hyst	Age	Year	Size	Freq^§^	LN^†^
25	(+)	TLH		2019		9.3	20.3
22	(+)	TLH		2017–2018		14.9	10.4
28	NOS		≥ 60	2019		1.3	9.1
19	(+)	TLH		2016		6.9	8.5
26	(+)	LAVH		2019		1.4	8.1
13	(−)	TLH	≥ 60			7.6	5.1
23	(+)	LAVH		2017–2018		3.1	4.8
20	(+)	LAVH		2016		1.7	3.3
27	NOS		≥ 60	2016–2018		3.7	2.8
14	(−)	TLH	40–59			17.1	2.7
16	(−)	LAVH			L	3.5	1.8
30	NOS	TLH	40–59			8.7	1.8
15	(−)	TLH	< 40*			3.2	1.4
12	NOS		< 40			3.4	0.6
17	(−)	LAVH			S/M	4.1	0.6
29	NOS	LAVH,TVH	40–59			4.0	**
4	(−)	TVH				3.8	0
18	(+)	TVH		2016		0.6	0
21	(+)	TVH		2017–2018		1.1	0
24	(+)	TVH		2019		0.5	0

Patterns are shown in descending order of lymph node rates

Size, hospital bed capacity, *L* large, *S/M* small and mid, *Freq* proportional frequency, *LN* lymph node evaluation at hysterectomy, *Hyst* hysterectomy type, *TLH* total laparoscopic hysterectomy, *LAVH* laparoscopy-assisted vaginal hysterectomy, *TVH* total vaginal hysterectomy, *NOS* not otherwise specified

^‡^Corresponding classification tree figure with terminal pattern numbers is shown in Supplementary Fig. S1. There were 20 unique patterns identified in the analysis based on histology type, hysterectomy type, age, year, and hospital bed capacity

^§^Proportional frequency of each pattern among the whole cohort

^†^Rate of lymph node evaluation at hysterectomy per each classification pattern

*Including unknown

**Small number suppressed per HCUP guidelines

Among 20 patterns, there were 5 patterns (33.8% of study population) in which the lymph node evaluation rates were higher than the cohort-level rate (> 5.7%), ranging from 8.1 to 20.3% and 4 of 5 patterns had endometrial hyperplasia with atypia (Table 3). There were 4 patterns (6.0% of study population) that no lymph node evaluation was performed. In all of these, TVH was the surgical modality and 3 of 4 patterns had endometrial hyperplasia with atypia. Ultimately, the absolute difference of lymph node evaluation rates between the highest and lowest patterns was 20.3%.

There were 19,654 patients who had surgery for endometrial hyperplasia with atypia, of which 2158 (11.0%) patients had lymph node evaluation at hysterectomy. The lymph node evaluation rate increased from 6.9 to 17.9% from 2016 to 2019 (*P* *trend* < 0.001; Fig. 1). In a multivariable analysis (Table 4), independent characteristics for lymph node evaluation were similar to the results of cohort-level analysis. Of those, surgery in 2019 (aOR compared 2016 2.77), urban non-teaching hospitals (aOR compared to rural 2.64) and urban teaching hospitals (aOR compared to rural 2.50), TLH (aOR compared to LAVH 2.47), and age ≥ 60 years (aOR compared to < 40 years 2.15) were the factors exhibited larger than twofold effect size.

**Table 4:** Sensitivity analysis (Atypia cases)

Characteristic	LN^†^	aOR (95%CI)	*P* value
Age (years)			
< 40	6.4	1 (reference)	
40–59	10.8	1.83 (1.47–2.28)	< 0.001
≥ 60	12.2	2.15 (1.70–2.70)	< 0.001
Year			
2016	6.9	1 (reference)	
2017	9.0	1.33 (1.14–1.54)	< 0.001
2018	8.7	1.23 (1.05–1.43)	0.010
2019	17.9	2.77 (2.42–3.17)	< 0.001
Primary expected payer			
Medicare	11.7	1 (reference)	
Medicaid	9.8	1.11 (0.89–1.38)	0.371
Private including HMO	10.8	1.03 (0.90–1.17)	0.693
Self-pay	11.6	1.21 (0.87–1.68)	0.256
No charge	**	2.46 (0.82–7.41)	0.110
Other	10.9	1.09 (0.78–1.54)	0.613
Unknown	0	n/a	0.998
Household income			
QT1 (lowest)	9.6	1 (reference)	
QT2	10.5	1.13 (0.98–1.31)	0.091
QT3	11.0	1.12 (0.96–1.30)	0.142
QT4 (highest)	12.4	1.28 (1.09–1.50)	0.002
Unknown	11.7	1.20 (0.78–1.87)	0.411
Patient location			
Large central metropolitan	10.2	0.76 (0.67–0.87)	< 0.001
Large fringe metropolitan	11.8	1 (reference)	
Medium metropolitan	11.6	1.03 (0.9–1.18)	0.683
Small metropolitan	11.4	1.10 (0.91–1.32)	0.331
Micropolitan	9.6	1.16 (0.95–1.42)	0.156
Not metropolitan or micropolitan	10.2	1.19 (0.95–1.49)	0.138
Unknown	**	0.66 (0.12–3.74)	0.638
Obesity			
No	10.3	1 (reference)	
Yes	12.1	1.13 (1.03–1.24)	0.012
Charlson comorbidity index			
0	10.7	1 (reference)	
1	11.4	1.01 (0.90–1.12)	0.926
2	11.0	0.96 (0.80–1.14)	0.619
≥ 3	13.2	1.02 (0.82–1.27)	0.852
Hospital bed capacity			
Small	6.1	1 (reference)	
Mid	9.5	1.07 (0.80–1.43)	0.640
Large	12.0	1.40 (1.04–1.87)	0.025
Hospital location/teaching			
Rural	3.8	1 (reference)	
Urban non-teaching	10.1	2.64 (1.85–3.77)	< 0.001
Urban teaching	11.7	2.50 (1.76–3.56)	< 0.001
Hospital region			
Northeast	8.9	0.69 (0.59–0.79)	< 0.001
Midwest	11.3	1.04 (0.93–1.18)	0.498
South	10.7	1 (reference)	
West	13.2	1.32 (1.15–1.51)	< 0.001
Hysterectomy modality			
TLH	12.9	2.47 (2.09–2.93)	< 0.001
LAVH	5.2	1 (reference)	
TVH	0	n/a	0.988

*aOR* adjusted odds ratio, *CI* confidence interval, *QT* quartile, *TLH* total laparoscopic hysterectomy, *LAVH* laparoscopy-assisted vaginal hysterectomy, *TVH* total vaginal hysterectomy

^†^LN rate (%) per row. A binary logistic regression model for multivariable analysis. All the listed covariates were entered in the modeling

In this atypia group, there were 16 unique patterns of lymph node evaluation at hysterectomy (Supplementary Fig. S2 and Table S2). Of those, patients who had TLH at urban centers in 2019 (22.6% of study population) had a lymph node evaluation rate of 20.9%. In contrast, none of patients who underwent TVH (5.6% of study population) had lymph node evaluation. Absolute difference for lymph node evaluation between the highest and lowest patterns was 20.9%.

Among 19,552 patients with endometrial hyperplasia without atypia, 492 (2.5%) patients had lymph node evaluation at the time of hysterectomy. The lymph node evaluation rate increased from 1.8 to 4.4% and from 2016 to 2019 (*P* *trend* < 0.001; Fig. 1). Independent factors for lymph node evaluation were also largely similar to the atypia cases (Supplementary Table S3). Among 12 unique patterns for nodal evaluation (Supplementary Fig. S3 and Table S4), patients aged ≥ 60 years who underwent TLH in 2019 (4.6% of study population) had the highest rate of lymph node evaluation (8.1%).

Last, trend of hysterectomy modality was examined (Supplementary Fig. S4). At the cohort level, the modality of hysterectomy is shifting toward TLH (14.0% relative increase), whereas LAVH (29.3% relative decrease) and TVH (34.0% relative decrease) significantly decreased during the 4-year study period (all, *P* *trend* < 0.001). These trends were similar in patients with atypia and those without atypia (Supplementary Fig. S4).

## Discussion

### Principal findings

Key results of this study are the following two. First, lymph node evaluation is increasingly incorporated into the surgical treatment of patients with endometrial hyperplasia, particularly those with atypical hyperplasia. Second, utilization of lymph node evaluation at hysterectomy for endometrial hyperplasia was markedly heterogeneous based on clinical characteristics and treatment-related factors.

### Results

The utilization rate of lymph node evaluation in this study of ambulatory surgical procedures appears to be similar to what was observed in the inpatient setting where the majority had abdominal hysterectomy during the same study period from 2016 to 2019 (ambulatory *versus* inpatient setting, 11.0% *versus* 9.4% for atypia, and 2.5% *versus* 2.8% for non-atypia) [11]. The aforementioned U.S. hospital-based study for atypical endometrial hyperplasia also reported similar utilization of lymph node evaluation from 2012 to 2018 (11.2%) [10]. All three large-scale analyses suggest increasing trends in lymph node evaluation [10, 11]. Collectively, despite the lack of prospective data, U.S. surgeons are gradually adopting this procedure in the surgical management of endometrial hyperplasia.

Surgeons are clearly taking the histologic type into account when considering lymph node evaluation for endometrial hyperplasia. This information was the primary indicator of lymph node evaluation (Supplementary Fig. S1), and endometrial hyperplasia with atypia had the largest odds for lymph node evaluation among the measured covariates (Table 2). This likely reflects concern for the increased risk of occult malignancy with this histologic type [4, 5].

While less frequent, nearly 2–3% of patients without atypia had lymph node evaluation at hysterectomy. This is of concern as the risk of occult endometrial cancer is much lower for patients with endometrial hyperplasia without atypia [4, 5]. Surgeon’s understanding and view of endometrial hyperplasia without atypia were not assessed in this study and merits further investigation.

Other identified factors for nodal evaluation such as older age and obesity are known risk factors for occult endometrial cancer that may trigger this surgical procedure [19]. Academic centers were more likely to perform this procedure. It is unknown if this is due to individual surgeon decision-making or hospital factors, such as the availability of near-infrared system for sentinel lymph node mapping.

Modality of hysterectomy for endometrial hyperplasia has been gradually shifting to TLH which was associated with increased utilization of lymph node evaluation. While the increasing trend of TLH is not endometrial hyperplasia specific and is also occurring for benign gynecologic disease [13], it is of interest to examine if this trend is influenced by surgeons’ desire to incorporate lymph node evaluation into the treatment of endometrial hyperplasia.

Based on patient, hospital, surgical, and histologic factors, the utilization of lymph node evaluation differed significantly. This was noted for both atypical hyperplasia and hyperplasia without atypia. Marked variability was noted for modality of hysterectomy comparing TLH and TVH. For instance, among the patients with atypia who had surgery in 2019, nearly one in 5 patients had lymph node evaluation among those who had TLH (20.4%), whereas none of those who had TVH had lymph node evaluation (Supplementary Fig. S2). Even among the gynecologic oncologists, opinions regarding the utility of nodal sampling for endometrial hyperplasia are variable [22–24]. Taken together, this diverse range of surgical practice of lymph node evaluation for endometrial hyperplasia implies the lack of universal consensus.

### Strengths and limitations

Strengths of our analysis include the large sample size, national-level analysis, and recent period of study. However, there are several limitations in this study. First, unmeasured bias is inherent to retrospective study. Possible confounders that were not captured in the study but may influence the analysis including the type of lymph node evaluation performed (sentinel lymph node biopsy or lymphadenectomy), preoperative diagnosis for surgery, surgeon type (gynecologic oncologist or gynecologist), shared decision-making process with patient, and use of robotic-assisted surgical system.

Second, to capture only patients without a diagnosis of endometrial cancer preoperatively, we excluded patients with a diagnosis of endometrial cancer from our cohort. In so doing we undoubtedly excluded some patients in whom endometrial cancer was only identified postoperatively. Our rates of nodal evaluation would likely have been higher if these patients could have been accurately identified and included in the analysis. Third, final pathologic information for occult endometrial cancer including incidence, histologic type, tumor differentiation, and cancer stage as well as lymph node metastasis in staged cases were not available in this study, but these are key outcome measures for this type of study. Likewise, quality-of-life metrics including long-term follow-up after surgery were not available in the NASS program. Third, accuracy of data in the NASS program was not assessable without actual medical record review. Last, generalizability in different study population is unknown.

### Conclusion

Lymph node evaluation at the time of minimally invasive hysterectomy for endometrial hyperplasia in the ambulatory surgery setting appears to be gradually increasing in the USA. Investigations have recently begun in the past few years [10, 11, 25, 26], and more studies are surely warranted to examine the benefits and risks of this surgical procedures. Barring more data to justify this surgical procedure, careful patient selection and balanced counseling are necessary.

## Supplementary Information

Below is the link to the electronic supplementary material.

Supplementary file1 (DOCX 551 KB)

## Data Availability

The data on which this study is based are publicly available upon request at Healthcare Cost and Utilization Project, Agency for Healthcare Research and Quality. https://www.hcup-us.ahrq.gov/nassoverview.jsp.

## Data transparency

The manuscript’s corresponding author (Koji Matsuo) affirms that the manuscript is an honest, accurate, and transparent account of the study being reported; that no important aspects of the study have been omitted; and that any discrepancies from the study as planned (and, if relevant, registered) have been explained.

## References

[1] Armstrong AJ, Hurd WW, Elguero S, Barker NM, Zanotti KM (2012). Diagnosis and management of endometrial hyperplasia. J Minim Invasive Gynecol.

[2] Trimble CL, Method M, Leitao M (2012). Management of endometrial precancers. Obstet Gynecol.

[3] Reed SD, Newton KM, Clinton WL (2009). Incidence of endometrial hyperplasia. Am J Obstet Gynecol.

[4] Kurman RJ, Kaminski PF, Norris HJ (1985). The behavior of endometrial hyperplasia. A long-term study of “untreated” hyperplasia in 170 patients. Cancer.

[5] Trimble CL, Kauderer J, Zaino R (2006). Concurrent endometrial carcinoma in women with a biopsy diagnosis of atypical endometrial hyperplasia: a Gynecologic Oncology Group study. Cancer.

[6] Costales AB, Schmeler KM, Broaddus R (2014). Clinically significant endometrial cancer risk following a diagnosis of complex atypical hyperplasia. Gynecol Oncol.

[7] Morice P, Leary A, Creutzberg C, Abu-Rustum N, Darai E (2016). Endometrial cancer. Lancet.

[8] Zaino RJ, Kauderer J, Trimble CL (2006). Reproducibility of the diagnosis of atypical endometrial hyperplasia: a Gynecologic Oncology Group study. Cancer.

[9] Vetter MH, Smith B, Benedict J (2020). Preoperative predictors of endometrial cancer at time of hysterectomy for endometrial intraepithelial neoplasia or complex atypical hyperplasia. Am J Obstet Gynecol.

[10] Dioun S, Chen L, Melamed A (2021). Uptake and outcomes of sentinel lymph node mapping in women with atypical endometrial hyperplasia. Obstet Gynecol.

[11] Matsuo K, Violette CJ, Mandelbaum RS, Tavakoli A, Klar M, Wright JD (2022). Increasing utilization of surgical nodal evaluation at hysterectomy for endometrial hyperplasia. Obstet Gynecol.

[12] Wright JD, Herzog TJ, Tsui J (2013). Nationwide trends in the performance of inpatient hysterectomy in the United States. Obstet Gynecol.

[13] Wright JD, Huang Y, Li AH, Melamed A, Hershman DL (2022). Nationwide estimates of annual inpatient and outpatient hysterectomies performed in the United States. Obstet Gynecol.

[14] Agency for Healthcare Research and Quality. Overview of the Nationwide Ambulatory Surgery Sample (NASS). https://www.hcup-us.ahrq.gov/nassoverview.jsp. Accessed 21 June 2022

[15] Matsuo K, Violette CJ, Mandelbaum RS (2022). Substantial variability in ovarian conservation at hysterectomy for endometrial hyperplasia. Am J Obstet Gynecol.

[16] Wright JD, Cham S, Chen L (2017). Utilization of sentinel lymph node biopsy for uterine cancer. Am J Obstet Gynecol.

[17] Polan RM, Rossi EC, Barber EL (2019). Extent of lymphadenectomy and postoperative major complications among women with endometrial cancer treated with minimally invasive surgery. Am J Obstet Gynecol..

[18] Agency for Healthcare Research and Quality. Clinical Classifications Software for Services and Procedures. https://www.hcup-us.ahrq.gov/toolssoftware/ccs_svcsproc/ccssvcproc.jsp. Accessed 21 June 2022

[19] Matsuo K, Ramzan AA, Gualtieri MR (2015). Prediction of concurrent endometrial carcinoma in women with endometrial hyperplasia. Gynecol Oncol.

[20] Charlson ME, Pompei P, Ales KL, MacKenzie CR (1987). A new method of classifying prognostic comorbidity in longitudinal studies: development and validation. J Chronic Dis.

[21] Ghaferi AA, Schwartz TA, Pawlik TM (2021). STROBE reporting guidelines for observational studies. JAMA Surg.

[22] Shalowitz DI, Goodwin A, Schoenbachler N (2019). Does surgical treatment of atypical endometrial hyperplasia require referral to a gynecologic oncologist?. Am J Obstet Gynecol.

[23] Sullivan MW, Philp L, Kanbergs AN (2021). Lymph node assessment at the time of hysterectomy has limited clinical utility for patients with pre-cancerous endometrial lesions. Gynecol Oncol.

[24] Touhami O, Gregoire J, Renaud MC, Sebastianelli A, Grondin K, Plante M (2018). The utility of sentinel lymph node mapping in the management of endometrial atypical hyperplasia. Gynecol Oncol.

[25] Mueller JJ, Rios-Doria E, Park KJ, Broach VA, Alektiar KM, Jewell EL, Zivanovic O, Sonoda Y, Abu-Rustum NR, Leitao MM, Gardner GJ (2023). Sentinel lymph node mapping in patients with endometrial hyperplasia: a practice to preserve or abandon?. Gynecol Oncol.

[26] Matanes E, Amajoud Z, Kogan L, Mitric C, Ismail S, Raban O, Knigin D, Levin G, Bahoric B, Ferenczy A, Pelmus M, Lecavalier-Barsoum M, Lau S, Salvador S, Gotlieb WH (2023). Is sentinel lymph node assessment useful in patients with a preoperative diagnosis of endometrial intraepithelial neoplasia?. Gynecol Oncol.

