# Correction: Lymph node evaluation for endometrial hyperplasia: a nationwide analysis of minimally invasive hysterectomy in the ambulatory setting

**DOI:** 10.1007/s00464-023-10137-3

**Published:** 2023-05-15

**Authors:** Koji Matsuo, Katharine M. Ciesielski, Rachel S. Mandelbaum, Matthew Y. W. Lee, Neda D. Jooya, Lynda D. Roman, Jason D. Wright

**Affiliations:** 1 Division of Gynecologic Oncology, Department of Obstetrics and Gynecology, University of Southern California, 2020 Zonal Avenue, IRD 520, Los Angeles, CA 90033 USA; 2 Norris Comprehensive Cancer Center, University of Southern California, Los Angeles, CA USA; 3 Division of Reproductive Endocrinology & Infertility, Department of Obstetrics and Gynecology, University of Southern California, Los Angeles, CA USA; 4 Division of Gynecologic Oncology, Department of Obstetrics and Gynecology, Columbia University College of Physicians and Surgeons, New York, NY USA

**Correction: Surgical Endoscopy** https://doi.org/10.1007/s00464-023-10081-2

The original online version of this article was revised to correct the presentation of the name of author Neda D. Jooya.

The original article has been corrected.

