# Correction: Lymph node evaluation for endometrial hyperplasia: a nationwide analysis of minimally invasive hysterectomy in the ambulatory setting

**DOI:** 10.1007/s00464-023-10174-y

**Published:** 2023-06-05

**Authors:** Koji Matsuo, Katharine M. Ciesielski, Rachel S. Mandelbaum, Matthew W. Lee, Neda D. Jooya, Lynda D. Roman, Jason D. Wright

**Affiliations:** 1 Division of Gynecologic Oncology, Department of Obstetrics and Gynecology, University of Southern California, 2020 Zonal Avenue, IRD 520, Los Angeles, CA 90033 USA; 2 Norris Comprehensive Cancer Center, University of Southern California, Los Angeles, CA USA; 3 Division of Reproductive Endocrinology & Infertility, Department of Obstetrics and Gynecology, University of Southern California, Los Angeles, CA USA; 4 Division of Gynecologic Oncology, Department of Obstetrics and Gynecology, Columbia University College of Physicians and Surgeons, New York, NY USA

**Correction to: Surgical Endoscopy** 10.1007/s00464-023-10081-2

The original online version of this article was revised to correct the presentation of one coauthor's name. The coauthor's correct name is Matthew W. Lee.

The original article has been corrected.

