# Supplementary material for: Lymph node evaluation for endometrial hyperplasia: a nationwide analysis of minimally invasive hysterectomy in the ambulatory setting
Source: Surg Endosc. 2023 May 8;37(8):6163–71. doi: 10.1007/s00464-023-10081-2 (PMC10338549; doi:10.1007/s00464-023-10081-2)
Supplement: Supplementary file 1 — Supplementary file1 (DOCX 551 KB) [file 464_2023_10081_MOESM1_ESM.docx]

**Supplemental Table S1. Coding information.**

| Characteristic | ICD-10-CM | CPT | CCS |
| --- | --- | --- | --- |
| Endometrial hyperplasia | N85.0 |  |  |
| Non-atypia | N85.01 |  |  |
| Atypia | N85.02 |  |  |
| NOS | N85.00 |  |  |
| Hysterectomy |  |  |  |
| TLH |  | 58548, 58570, 58571, 58572, 58573, 58575 |  |
| LAVH |  | 58550, 58552, 58553, 58554, 58553 |  |
| TVH |  | 58260, 58262, 58263, 58267, 58270, 58275, 58290, 58291, 58292, 58293, 58294, 58285 |  |
| Lymph node evaluation |  | 38500, 38562, 38564, 38570, 38571, 38572, 38573, 38589, 38770, 38780, 38790, 38792, 38900, 57109, 57531, 58200, 58210, 58548, 58943, 58951, 58954, 58958, 58960, 78195, 78800, 78801 | 67 |

Abbreviations: ICD-10-CM, International Classification of Disease, 10th revision, Clinical Modification; CPT, Current Procedural Terminology; CCS, Clinical Classification Software; TLH, total laparoscopic hysterectomy; LAVH, laparoscopy-assisted vaginal hysterectomy; and TVH, total vaginal hysterectomy.

**Supplemental Table S2. Classification-tree model for nodal evaluation (atypia cases).**

| Pattern^‡^ | Year | Hyst | Size | Payer | Area | Setting | Freq^§^ | LN^†^ |
| --- | --- | --- | --- | --- | --- | --- | --- | --- |
| 22 | 2019 | TLH |  |  |  | Urban | 22.6 | 20.9 |
| 19 | 2017-18 | TLH |  |  | W |  | 6.4 | 12.8 |
| 18 | 2017-18 | TLH |  |  | MW,S |  | 24.1 | 10.6 |
| 24 | 2019 | LAVH |  |  |  | Urban | 3.1 | 9.3 |
| 13 | 2016 | TLH | M/L |  |  |  | 16.7 | 8.8 |
| 23 | 2019 | TLH |  |  |  | Rural | 1 | 7.9 |
| 17 | 2017-18 | TLH |  |  | NE |  | 7.2 | 7.2 |
| 15 | 2016 | LAVH |  | Mcr/Mcd |  |  | 1.3 | 6.3 |
| 20 | 2017-18 | LAVH | M/L |  |  |  | 7.1 | 5.4 |
| 14 | 2016 | TLH | Sm |  |  |  | 0.8 | ** |
| 16 | 2016 | LAVH |  | Othr |  |  | 2.9 | 1.9 |
| 5 | 2016 | TVH |  |  |  |  | 1.6 | 0 |
| 8 | 2017-18 | TVH |  |  |  |  | 2.8 | 0 |
| 11 | 2019 | TVH |  |  |  |  | 1.2 | 0 |
| 21 | 2017-18 | LAVH | Sm |  |  |  | 0.8 | 0 |
| 25 | 2019 | LAVH |  |  |  | Rural | 0.4 | 0 |

Patterns are shown in descending order of lymph node rates. ‡ Corresponding classification-tree figure with terminal pattern numbers is shown in Supplemental Figure S2. There were 16 unique patterns identified in the analysis based on year, hysterectomy type, hospital bed capacity, primary payer, region, and teaching setting. § Proportional frequency of each pattern among the whole cohort. † Rate of lymph node evaluation at hysterectomy per each classification pattern. **small number suppressed per HCUP guidelines. Abbreviations: Freq, proportional frequency of each pattern; LN, lymph node evaluation at hysterectomy; Hyst, hysterectomy type; TLH, total laparoscopic hysterectomy; LAVH, laparoscopy-assisted vaginal hysterectomy; and TVH, total vaginal hysterectomy; NOS, not otherwise specified; Size, hospital bed capacity; M/L, mid and large; Sm, small; Payr, primary expected payer; Mcr, Medicare; Mcd, Medicaid; Othr, other; NE, Northeast; MW, Midwest; S, South; W, West; Setting, hospital location / teaching setting; Urb, urban teaching and urban non-teaching.

**Supplemental Table S3. Sensitivity analysis (Non-atypia cases).**

| Characteristic | LN^†^ | aOR (95%CI) | *P*-value |
| --- | --- | --- | --- |
| **Age** |  |  |  |
| <40 | 1.1 | 1 (reference) |  |
| 40-59 | 2.1 | 1.74 (1.14-2.66) | 0.011 |
| ≥60 | 4.0 | 2.81 (1.79-4.40) | <0.001 |
| Unknown | ** |  |  |
| **Year** |  |  |  |
| 2016 | 1.8 | 1 (reference) |  |
| 2017 | 2.1 | 1.12 (0.85-1.47) | 0.431 |
| 2018 | 2.1 | 1.05 (0.79-1.40) | 0.754 |
| 2019 | 4.4 | 2.28 (1.77-2.94) | <0.001 |
| **Primary expected payer** |  |  |  |
| Medicare | 4.0 | 1 (reference) |  |
| Medicaid | 1.6 | 0.66 (0.42-1.03) | 0.066 |
| Private including HMO | 2.3 | 0.80 (0.62-1.03) | 0.079 |
| Self-pay | ** | 0.61 (0.25-1.48) | 0.276 |
| No charge | 0 | n/a | 0.998 |
| Other | ** | 0.78 (0.39-1.58) | 0.488 |
| Unknown | ** | 3.67 (0.64-21.03) | 0.144 |
| **Household income** |  |  |  |
| QT1 (lowest) | 2.3 | 1 (reference) |  |
| QT2 | 2.3 | 1.00 (0.76-1.31) | 0.971 |
| QT3 | 2.2 | 0.77 (0.58-1.03) | 0.074 |
| QT4 (highest) | 3.4 | 1.06 (0.79-1.43) | 0.702 |
| Unknown | 4.0 | 1.70 (0.88-3.28) | 0.111 |
| **Patient location** |  |  |  |
| Large central metropolitan | 3.2 | 0.83 (0.65-1.06) | 0.136 |
| Large fringe metropolitan | 3.1 | 1 (reference) |  |
| Medium metropolitan | 2.2 | 0.68 (0.52-0.89) | 0.006 |
| Small metropolitan | 2.3 | 0.91 (0.64-1.29) | 0.589 |
| Micropolitan | 1.2 | 0.76 (0.48-1.20) | 0.232 |
| Not metropolitan or micropolitan | 1.6 | 0.86 (0.54-1.39) | 0.538 |
| Unknown | 0 | n/a | 0.998 |
| **Obesity** |  |  |  |
| No | 2.2 | 1 (reference) |  |
| Yes | 3.2 | 1.22 (1.01-1.47) | 0.047 |
| **Charlson comorbidity index** |  |  |  |
| 0 | 2.3 | 1 (reference) |  |
| 1 | 2.6 | 0.97 (0.76-1.22) | 0.768 |
| 2 | 4.0 | 1.31 (0.97-1.78) | 0.079 |
| ≥3 | 3.6 | 0.96 (0.59-1.54) | 0.850 |
| **Hospital bed capacity** |  |  |  |
| Small | 0.8 | 1 (reference) |  |
| Mid | 1.5 | 0.90 (0.52-1.56) | 0.717 |
| Large | 3.5 | 1.89 (1.10-3.26) | 0.021 |
| **Hospital location / teaching** |  |  |  |
| Rural | ** | 1 (reference) |  |
| Urban non-teaching | 1.7 | 4.07 (1.66-10.01) | 0.002 |
| Urban teaching | 3.2 | 4.94 (2.01-12.10) | <0.001 |
| **Hospital region** |  |  |  |
| Northeast | 2.7 | 0.83 (0.64-1.08) | 0.168 |
| Midwest | 1.8 | 0.74 (0.58-0.95) | 0.018 |
| South | 2.7 | 1 (reference) |  |
| West | 3.4 | 1.44 (1.10-1.87) | 0.007 |
| **Hysterectomy modality** |  |  |  |
| TLH | 3.2 | 2.49 (1.81-3.42) | <0.001 |
| LAVH | 1.1 | 1 (reference) |  |
| TVH | 0 | n/a | 0.985 |

† LN rate (%) per row. A binary logistic regression model for multivariable analysis. All the listed covariates were entered in the modeling. Abbreviations: aOR, adjusted odds ratio; CI, confidence interval; QT, quartile; TLH, total laparoscopic hysterectomy; LAVH, laparoscopy-assisted vaginal hysterectomy; TVH, total vaginal hysterectomy; and NOS, not otherwise specified.

**Supplemental Table S4. Classification-tree model for nodal evaluation (non-atypia cases).**

| Pattern^‡^ | Hyst | Age | Size | Year | Setting | CCI | Freq^§^ | LN^†^ |
| --- | --- | --- | --- | --- | --- | --- | --- | --- |
| 10 | TLH | ≥60 |  | 2019 |  |  | 4.6 | 8.1 |
| 17 | LAVH |  | L | 2019 |  |  | 1.6 | 4.4 |
| 14 | TLH | <40* |  |  |  | 2 | 0.5 | ** |
| 9 | TLH | ≥60 |  | <2019 |  |  | 14.8 | 4.2 |
| 11 | TLH | 40-59 |  |  | Urb-T |  | 29.7 | 3.4 |
| 13 | TLH | 40-59 |  |  | Urb-nT |  | 10.1 | 1.8 |
| 18 | LAVH | ≥60 | S/M |  |  |  | 2.5 | ** |
| 16 | LAVH |  | L | <2019 |  |  | 7.3 | 1.2 |
| 15 | TLH | <40* |  |  |  | <2,≥3 | 7.7 | 1.1 |
| 12 | TLH | 40-59 |  |  | Rural |  | 3.7 | ** |
| 19 | LAVH | <60 | S/M |  |  |  | 7.9 | ** |
| 1 | TVH |  |  |  |  |  | 9.7 | 0 |

Patterns are shown in descending order of lymph node rates. ‡ Corresponding classification-tree figure with terminal pattern numbers is shown in Supplemental Figure S3. There were 12 unique patterns identified in the analysis based on hysterectomy type, age, hospital bed capacity, year, teaching setting, and comorbidity. § Proportional frequency of each pattern among the whole cohort. † Rate of lymph node evaluation at hysterectomy per each classification pattern. **small number suppressed per HCUP guidelines. Abbreviations: Freq, proportional frequency of each pattern; LN, lymph node evaluation at hysterectomy; Hyst, hysterectomy type; TLH, total laparoscopic hysterectomy; LAVH, laparoscopy-assisted vaginal hysterectomy; and TVH, total vaginal hysterectomy; Size, hospital bed capacity; L, large; S/M, small and mid; Setting, hospital location / teaching setting; Urb-T, urban teaching; Urb-nT, urban non-teaching; and CCI, Charlson comorbidity index.

**Supplemental Figure S1. Classification-tree model (all cases).**


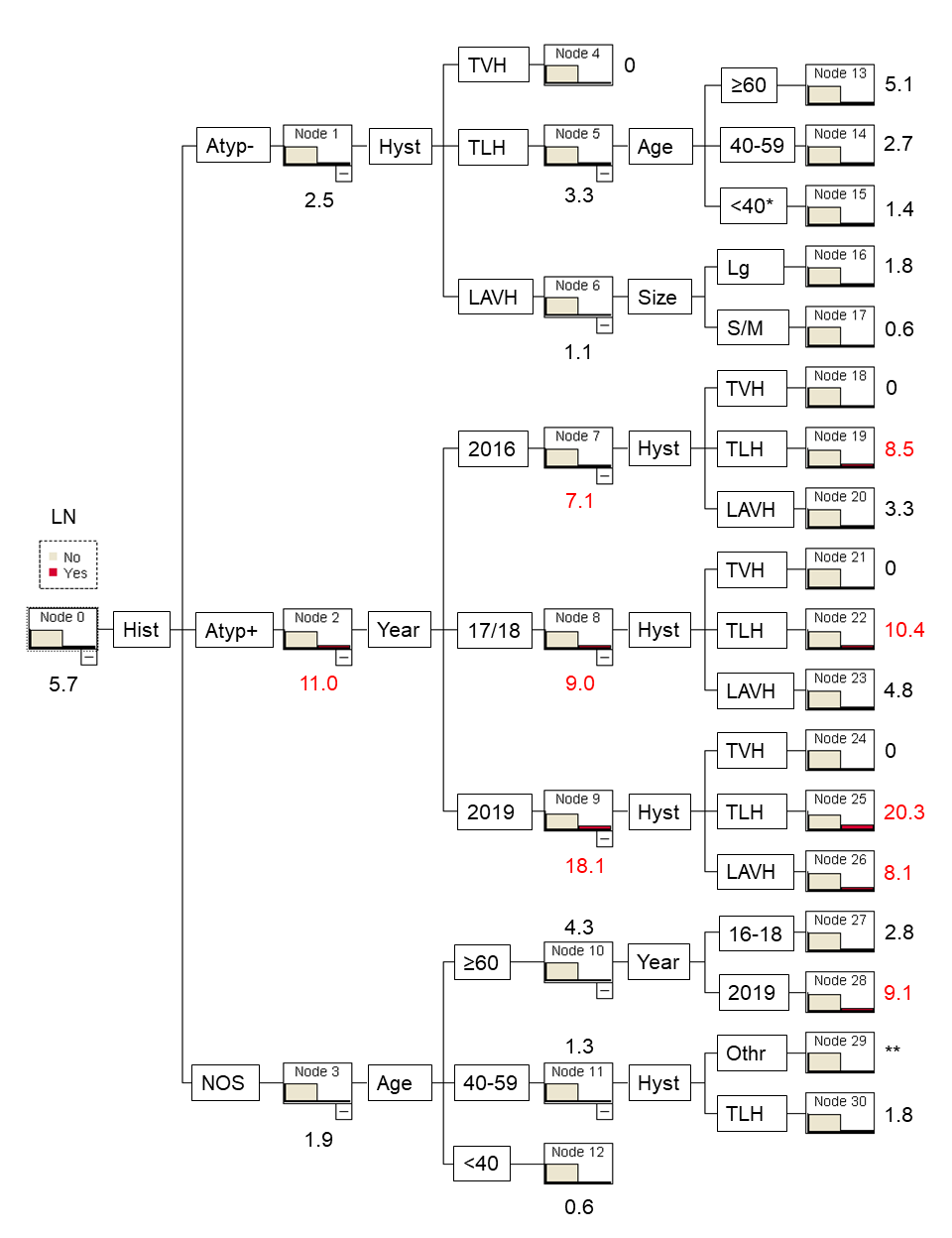


Lymph node evaluation rates are shown in each cell. The rates higher than average (5.7%) are highlighted with red. Metadata is shown in Table 4. *including unknown. **small number suppressed per HCUP guidelines. Abbreviations: Freq, proportional frequency of each pattern; LN, lymph node evaluation at hysterectomy; Hyst, hysterectomy type; TLH, total laparoscopic hysterectomy; LAVH, laparoscopy-assisted vaginal hysterectomy; and TVH, total vaginal hysterectomy; NOS, not otherwise specified; Size, hospital bed capacity; Lg, large; S/M, small and mid; Payr, primary expected payer; Mcr, Medicare; Mcd, Medicaid; Othr, other; NE, Northeast; MW, Midwest; S, South; W, West; Setting, hospital location / teaching setting; Urb, urban teaching and urban non-teaching.

**Supplemental Figure S2. Classification-tree model (atypia type).**


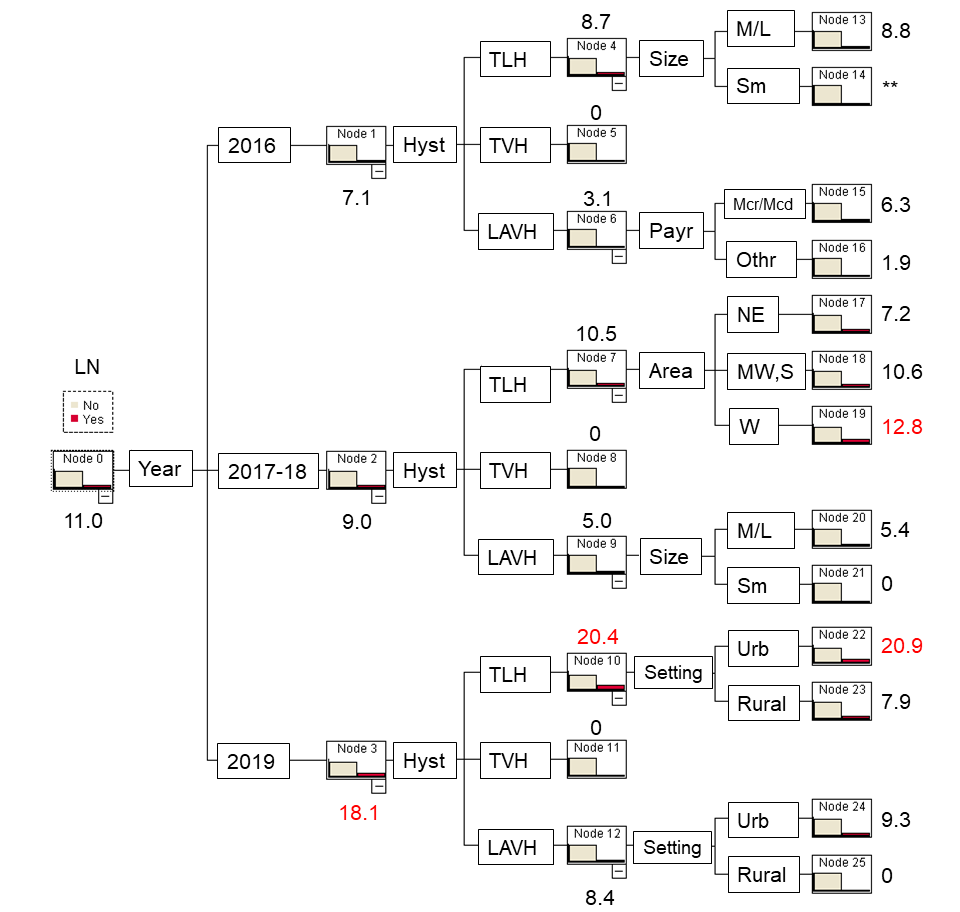


Lymph node evaluation rates are shown in each cell. The rates higher than average (11.0%) are highlighted with red. Metadata is shown in Supplemental Table S2. **small number suppressed per HCUP guidelines. Abbreviations: LN, lymph node evaluation at hysterectomy; Hyst, hysterectomy type; TLH, total laparoscopic hysterectomy; LAVH, laparoscopy-assisted vaginal hysterectomy; and TVH, total vaginal hysterectomy; NOS, not otherwise specified; Size, hospital bed capacity; Sm, small; M/L, mid and large; Payr, primary expected payer; Mcr, Medicare; Mcd, Medicaid; Othr, other; NE, Northeast; MW, Midwest; S, South; W, West; Setting, hospital location / teaching setting; Urb, urban teaching and urban non-teaching.


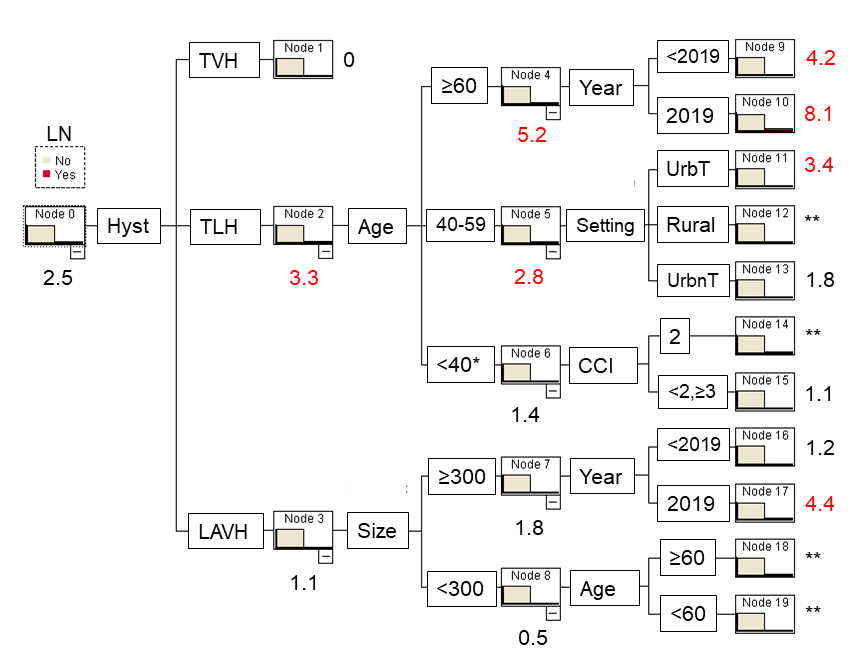
**Supplemental Figure S3. Classification-tree model (non-atypia type).**

Lymph node evaluation rates are shown in each cell. The rates higher than average (2.5%) are highlighted with red. Metadata is shown in Supplemental Table S3. *including unknown. **small number suppressed per HCUP guidelines. Abbreviations: LN, lymph node evaluation at hysterectomy; Hyst, hysterectomy type; TLH, total laparoscopic hysterectomy; LAVH, laparoscopy-assisted vaginal hysterectomy; and TVH, total vaginal hysterectomy; NOS, not otherwise specified; Size, hospital bed capacity; CCI, Charlson comorbidity index; Setting, hospital location / teaching setting; UrbT, urban teaching, UrbnT urban non-teaching.

**
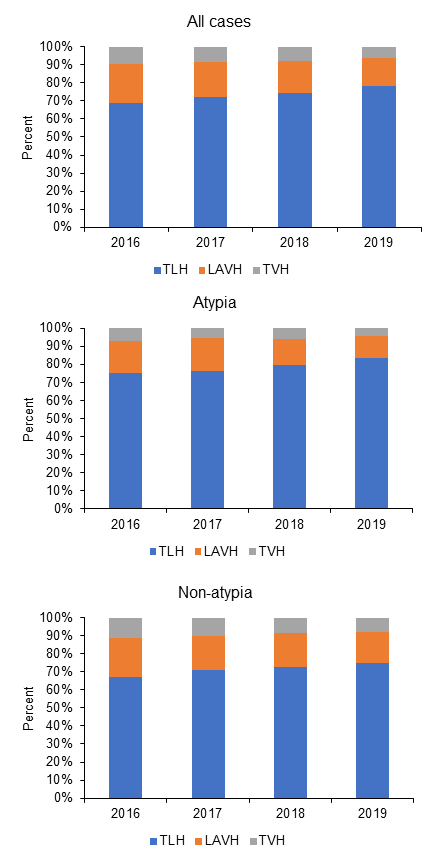
Supplemental Figure S4. Trends in hysterectomy type for endometrial hyperplasia.**

Proportional rates of minimally invasive hysterectomy types (TLH, LAVH, and TVH) per year are shown from 2016-2019 stratified by histology type (all cases, top panel; atypia, mid panel, and non-atypia, bottom panel). In all cases, the number of TLH cases increased from 68.8% to 78.4% (14.0% relative-increase) whereas LAVH cases from 21.5% to 15.2% (29.3% relative-decrease) and TVH cases from 9.7% to 6.4% (34.0% relative-decrease) from 2016-2019 (all, *P-trend*<0.001). In the atypia cases, the number of TLH cases increased from 75.1% to 83.3% (10.9% relative-increase) whereas LAVH cases from 17.9% to 12.4% (30.7% relative-decrease) and TVH cases from 6.9% to 4.3% (37.7% relative-decrease) from 2016-2019 (all, *P-trend*<0.001). This trend was also similar in the non-atypia group (TLH 66.9% to 75.1% [12.3% relative-increase]; LAVH 21.8% to 16.8% [22.9% relative-decrease]; and TVH 11.2% to 8.1% [27.7% relative-decrease]) (all, *P-trend*<0.001). Abbreviations: TLH, total laparoscopic hysterectomy; LAVH, laparoscopy-assisted vaginal hysterectomy; and TVH, total vaginal hysterectomy.
